# TPGS Decorated Liposomes as Multifunctional Nano-Delivery Systems

**DOI:** 10.1007/s11095-022-03424-6

**Published:** 2022-11-14

**Authors:** Muhammad Asim Farooq, Natalie L. Trevaskis

**Affiliations:** Drug Delivery, Disposition and Dynamics, Monash Institute of Pharmaceutical Sciences, Monash University, 399 Royal Parade, Parkville, VIC 3052 Australia

**Keywords:** cancer therapy, D-alpha-tocopheryl polyethylene glycol succinate (TPGS), liposomes, drug delivery, pharmacokinetics

## Abstract

Liposomes are sphere-shaped vesicles that can capture therapeutics either in the outer phospholipid bilayer or inner aqueous core. Liposomes, especially when surface-modified with functional materials, have been used to achieve many benefits in drug delivery, including improving drug solubility, oral bioavailability, pharmacokinetics, and delivery to disease target sites such as cancers. Among the functional materials used to modify the surface of liposomes, the FDA-approved non-ionic surfactant D-alpha-tocopheryl polyethylene glycol succinate (TPGS) is increasingly being applied due to its biocompatibility, lack of toxicity, applicability to various administration routes and ability to enhance solubilization, stability, penetration and overall pharmacokinetics. TPGS decorated liposomes are emerging as a promising drug delivery system for various diseases and are expected to enter the market in the coming years. In this review article, we focus on the multifunctional properties of TPGS-coated liposomes and their beneficial therapeutic applications, including for oral drug delivery, vaccine delivery, ocular administration, and the treatment of various cancers. We also suggest future directions to optimise the manufacture and performance of TPGS liposomes and, thus, the delivery and effect of encapsulated diagnostics and therapeutics.

## Introduction

Liposomes are versatile nanocarriers that have gained attention for multiple applications in the drug delivery, cosmetic, and food industries [1–3]. Liposomes were the first nano-drug delivery system approved by the United States Food and Drug Administration (FDA) in 1995 when liposomes encapsulating doxorubicin (Doxil®) were approved for ovarian cancer therapy [4]. The FDA has subsequently approved several other liposome-based formulations for cancer therapy, including liposomal formulations of daunorubicin (DaunoXome®) and vincristine (Marqibo®) [5]. Liposomal formulations of amphotericin B (e.g., Ambisome ®) have also proven an important development for the treatment of fungal infections. Liposomes are a new alternative for delivering vaccines such as the products Epaxal ® and Inflexal® V [6].

Liposomes are sphere-shaped vesicles containing a phospholipid bilayer and aqueous core. Hydrophilic molecules can be encapsulated in the aqueous core, whereas lipophilic molecules can be entrapped in the lipid bilayer. Therefore, this lipid-based carrier’s amphiphilic nature is suitable for loading therapeutic agents with a range of physicochemical properties [7]. Liposomes are also generally non-toxic as they are prepared with biocompatible lipids. Importantly, the properties of liposomes, such as their composition, size, surface charge, and modifications, can be purposely modified to control the pharmacokinetics and biodistribution of encapsulated therapeutics and other molecules. This has, in particular, led to the use of liposomes to prolong the half-life and alter the biodistribution of therapeutics. Importantly, this can improve drug safety and efficacy profiles, as has been demonstrated for several important anti-cancer drugs [8].

In the early days of liposome development, conventional liposomes were prepared without significant surface modifications. Early liposomes were associated with various limitations, including instability, inadequate drug loading, rapid drug release, and short blood circulation half-life [9]. The short half-life was found to result from rapid clearance from the blood circulation by the reticuloendothelial system in the liver and spleen following opsonisation (coating of the surface of the liposomes by opsonins in the blood) and subsequent recognition and removal by phagocytic cells [10]. A breakthrough in liposome development in the 1980s was the invention of ‘stealth liposomes’ with a surface coating that prevented opsonisation and phagocytosis, thus significantly prolonging the circulation half-life [11]. The first coating used to prevent opsonisation and prolong circulation half-life was the innovation of modifying liposomes’ surface by adding lipid conjugated polyethylene glycol (PEG) molecules [12].

In addition to PEG, the surface of liposomes has been modified with molecules such as carbohydrates [13], aptamers [14], peptides [15], polysaccharides [16], and vitamins [17]. Mostly these molecules are recognized by surface receptors on specific cells leading to ‘targeted’ delivery. These additions on the surface of liposomes have considerably heightened the application of liposomes in drug delivery, particularly for cancer diagnosis and therapy [18].

**Fig. 1:**
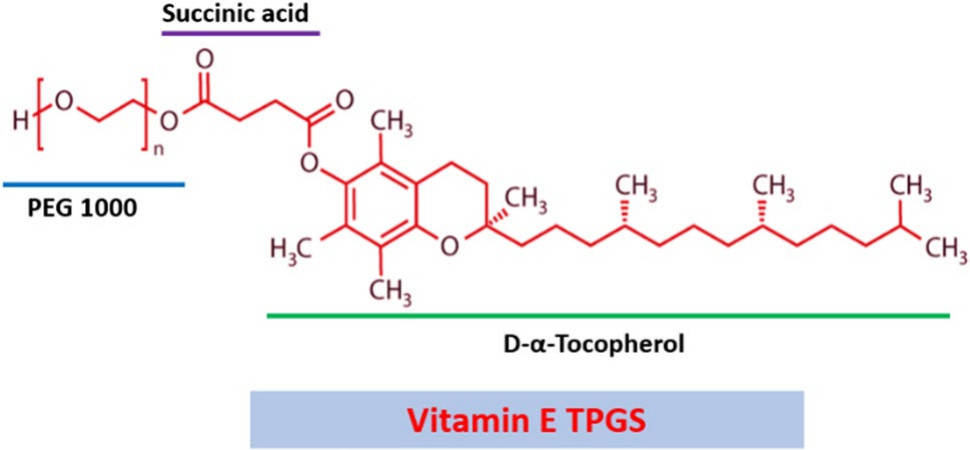
Chemical structure of Vitamin E TPGS.

An alternate form of PEGylation is the surface modification of liposomes with D-a-tocopheryl PEG 1000 succinate (vitamin E TPGS) [9]. (Fig. 1). As seen for PEG conjugated lipids, surface modification with TPGS can alter the pharmacokinetics (e.g., prolong the circulation half-life) and facilitate enhanced delivery to disease target sites [19–21]. In this review, we summarise the general properties of TPGS that make it a valuable molecule for surface engineering of liposomes. We provide an overview of studies demonstrating beneficial therapeutic applications of TPGS decorated liposomes, including to enhance oral and ocular drug delivery, vaccine delivery, and the treatment of various cancers. Finally, we discuss future ways in which TPGS liposomes may be exploited to advance health care.

## Properties of TPGS Relevant to Drug Delivery

TPGS, a non-ionic surfactant, has been widely used in formulations and delivery systems to enhance drug emulsification, solubilization, stability, and penetration [22]. TPGS is a safe pharmaceutical excipient approved for human use by the United States Food and Drug Administration (FDA). TPGS is synthesized via esterification of vitamin E succinate with a PEG chain [23, 24] (Fig. 1). TPGS has a waxy nature, a melting point between 37–41℃, hydrophilic-lipophilic balance (HLB) of 13.2, and critical micelle concentration (CMC) of 0.02 mM [25].

TPGS has been administered via most drug delivery routes, including oral, nasal, topical, and parenteral. A wide range of TPGS-coated nanocarriers has also been reported in the literature, including liposomes [26], nanoemulsions [27, 28], solid lipid nanoparticles (NPs) [29, 30], liquid crystalline NPs [31, 32] and micelles [33]. Functionalising nano-carriers with TPGS has been shown to improve cellular uptake (e.g., into various cancer cells), enhance permeability across cells, thus enhancing oral bioavailability, and extend the blood circulation half-life of valuable drug molecules *in vivo* [34, 35].

TPGS has several distinguishing features and advantages over PEG for drug delivery. An important feature associated with TPGS and not PEG is the ability of TPGS to act as a synergistic agent to reverse multidrug resistance (MDR) and inhibit P-glycoprotein (P-gp) mediated efflux of drugs such as anti-cancer agents [36, 37]. TPGS has additionally been used to form prodrugs designed to promote drug delivery to tumours [38]. Prodrugs typically display low or no pharmacological activity but can undergo a series of *in vivo* biotransformation/s to generate a parent drug with pharmacological activity [39]. The hydroxyl functional group on PEG in the TPGS structure is very reactive and has been conjugated with various cancer therapeutics to form TPGS prodrugs, including with doxorubicin, paclitaxel, gemcitabine, and cantharidin [40–43]. In TPGS-based prodrugs, TPGS usually acts as a drug carrier and P-gp inhibitor to reverse MDR in cancer therapy [44]. TPGS also has antioxidant activity [45] not seen with PEG, which can protect against the oxidative degradation of drugs in formulations during storage, thus enhancing the stability of formulations [46]. Similarly, as an antioxidant agent, TPGS has been used to develop many ocular formulations to prevent oxidative stress accompanying ophthalmic diseases such as glaucoma and cataracts [47].

**Fig. 2:**
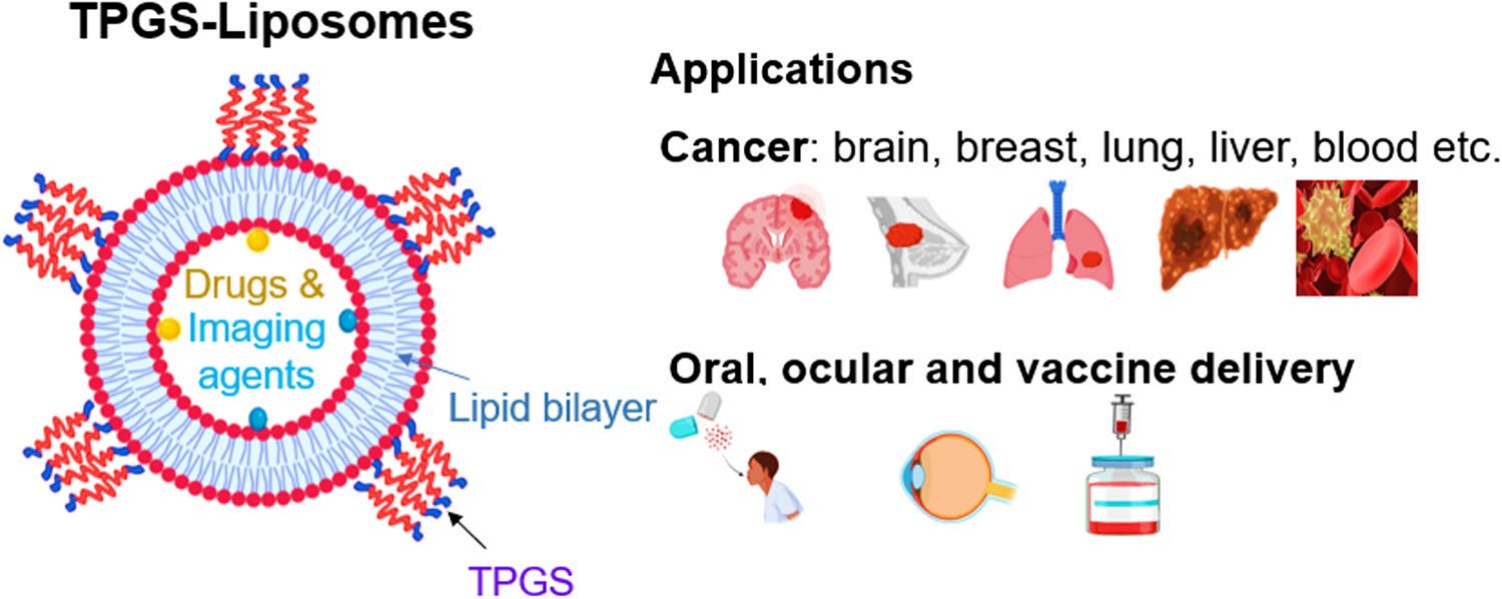
Schematic illustration of TPGS-coated liposomes as a multifunctional nanocarrier used for a variety of applications. Created with the assistance of BioRender.com.

Due to the multiple desirable properties of TPGS, TPGS-modified liposomes have been used for various applications that are summarised in Fig. 2 below. Table I also provides a detailed summary of studies using TPGS-liposomes to treat various diseases. Below we summarise the various applications of TPGS-liposomes.

**Table I:** Summary of Studies Reporting Therapeutic Benefits of TPGS-Modified Liposomes

No	Payload	Particle Size (nm)	Zeta Potential (mV)	%Encapsulation Efficiency	Route of Administration	Cell line/Animal Model	Major Findings	Ref
Brain cancer								
1	Resveratrol	61.8 ± 3.49	1.76 ± 0.83	84.59 ± 5.75	i.v	C6 glioma cells/ Charles foster rats	Higher entrapment efficiency of the drug, higher cytotoxicity and prolonged systemic circulation compared with unmodified liposomes	[93]
2	Docetaxel (DTX)	191.1 ± 1.5	-2.41 ± 0.30	64.10 ± 0.57	**-**	C6 glioma cells	Showed better liposomal stability and exhibited higher cellular uptake and *in vitro* cytotoxicity than conventional liposomes	[53]
3	DTX	80	-15.8	85	i.v	C6 glioma cells/ Male kunming mice	Excellent *in vitro* anti-cancer activity and higher brain biodistribution in mice compared to non-TPGS liposomes	[95]
4	Paclitaxel/ Artemether	90	-	-	i.v	Glioma C6 cells/ Male Sprague Dawley (SD) rats	Enhanced disruption of brain glioma vasculogenic mimicry (VM) channels compared to the free drug solution	[98]
5	Rginine-glycine-aspartic acid DTX / Quantum Dots (QDs)	182.3 ± 7.5	1.10 ± 0.25	68.45 ± 0.57	i.v	Charles foster rats	TPGS-modified liposomes improved DTX delivery into brain tissues and enhanced drug transportation across the BBB	[99]
Breast cancer								
6	Tyroservatid/Paclitaxel (PTX)	140	Neutral	86.79 ± 0.07	i.v	MB-231 cells/ Nude mice	Minimized the cytotoxicity of PTX and enhanced chemotherapeutic efficacy compared to free drug solution	[110]
7	DTX/QDS	210.5 ± 0.6	-6.27 ± 0.78	54.18 ± 0.62	-	MCF-7 cells	Showed controlled drug release and exhibited higher cellular uptake and cytotoxicity than non-targeting liposomes	[111]
8	PTX	282.6 ± 20.41	-17	40.89	-	MCF-7 and MCF-7/ADR cells	Supported sustained *in vitro* release and boosted cellular uptake compared to conventional liposomes	[112]
9	PTX/ lonidamine	120	34	-	i.v	MCF-7 and MCF-7/ADR cells/ Sprague dawley rats	Excellent drug accumulation at the tumour site and better cell uptake was observed than in a non-TPGS formulation	[113]
10	DTX/Trastuzumab	185.6 ± 2.42.4	-27.4 ± 0.50	49.10 ± 0.55	i.v	SK-BR-3 cells/ Sprague dawley rats	Improved the half-life of encapsulated drugs compared to the free drug solutions	[52]
11	Vorinostat	176.99 ± 2.06	-26.3 ± 0.8	83.23 ± 0.03	-	MCF-7 cells	Enhanced the solubility of vorinostat, *in vitro* cytotoxicity and cell uptake than unmodified liposomes	[22]
Lung cancer								
12	Luteolin (LUT)	176.2 ± 10.4	-23.55 ± 0.54	71.55 ± 0.26	i.v	A549 cells/ BALB/c nude mice	LUT-TPGS liposomes provided higher cellular uptake and cytotoxic effect than the free drug solution LUT-TPGS liposomes had extended circulation time and preferential accumulation at the tumour site than free luteolin solution	[120]
13	Ginsenoside	119.3 ± 1.4	-1.9 ± 0.4	98.4 ± 2.3	i.v	A549 cells / Male athymic nude mice	Enhanced the cellular uptake and cell cytotoxicity and displayed higher anti-tumor efficiency than free drug molecules	[121]
14	DTX	140.9 ± 6.0	-		i.v	A549 cells /Nude mice	Significantly enhanced the cell uptake in A549 cells and inhibited P-gp pumps more than the free drug	[122]
Liver cancer								
15	DOX/ siRNA	209.9 ± 2.8	12.5 ± 1.5	95.8 ± 1.3	s.c	H22 cells/ BALB/c female mice	Enhanced cellular uptake, promoted DOX diffusion, and increased DOX accumulation at tumour sites compared to free DOX solution	[127]
16	DOX/Bcl-2 siRNA	192.4 ± 2.8	12.7 ± 2.1	95.1 ± 1.9	i.v	Bel7402, Bel7402/5-/ Male BALB/c nude mice	TPGS inhibited P-gp efflux, boosted intracellular DOX concentration, and improved the anticancer effect of DOX	[129]
17	Artesunate	126.7 ± 9.9	-10.1 ± 1.43	78.8 ± 1.89	i.v	Male wistar rats	Enhanced the encapsulation efficiency, stability, circulation time and liver targeting compared to free drug	[132]
Blood cancer								
18	Emodin	121.1 ± 44.9	-13.1 ± 2.7	95.2% ± 3.0%,	i.v	L1210, K562 cells/ Imprinting control region mice (ICR)	Improved blood circulation of emodin compared to the free drug solution	[55]
TPGS liposomes for other applications								
Oral bioavailability								
19	Isoliquiritigenin	23.8 ± 0.9	-38.48 ± 0.29	97.33 ± 0.40,	Oral	ICR mice	Increased water solubility, oral bioavailability, and liver targeting of isoliquiritigenin compared to free drug solution	[24]
20	Syringic acid	40.01 ± 0.48	-38.50 ± 0.05	96.48 ± 0.76	Oral	Sprague dawley rats	Higher oral bioavailability and liver accumulation of syringic acid was seen after administration in TPGS-liposomes	[154]
21	6-Shogaol	23.50 ± 0.09	-	95.18 ± 0.28	Oral	Sprague dawley rats	Prolonged blood circulation and brain targeting compared to conventional liposomes	[157]
Ocular delivery								
22	Brinzolamide (Brz)	96.87 ± 4.43	-1.17 ± 1.91	95.41 ± 3.03	Ocular	Murine fibroblast L929 cells/ New Zealand rabbits	Showed sustained-release performance, and TPGS liposomes improved the penetration of Brz across the cornea compared to conventional liposomes	[168]
Vaccine delivery								
23	Ovalbumin	93.6 ± 0.9	29.9 ± 2.2	-	Nasal	L-132 cells/ Female C57/BL6 mice	Improved stability and successive delivery of antigen encapsulated in modified liposomes compared to free antigen delivery	[181]

## Preparation of TPGS-liposomes

**Fig. 3:**
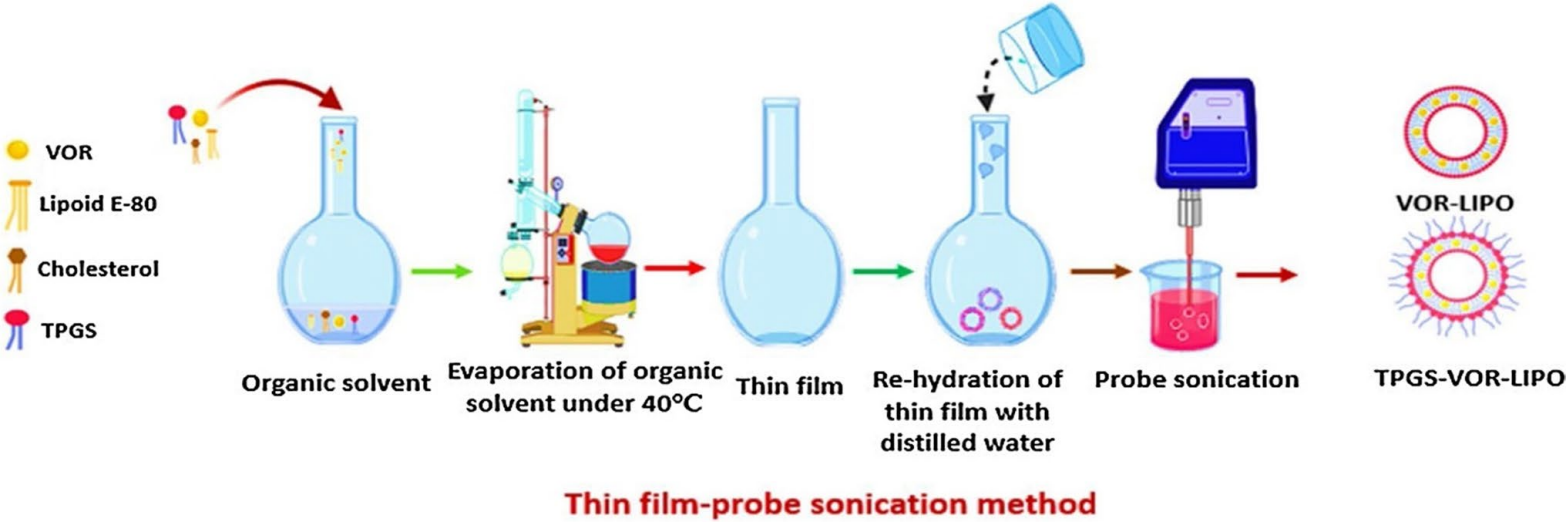
Schematic illustration of the preparation process of vorinostat liposomes (VOR-LIPO) and TPGS-VOR-LIPO [22]. Reprinted and adopted with permission from Elsevier Ltd. through Copyright Clearance Centre.

Different techniques are utilized to fabricate TPGS-modified liposomes, depending upon the desired formulation. These techniques overcome the hydrophobic nature of lipids when mixed with water. The main methods used included the thin film hydration method [22, 48–51], as shown in Fig. 3 [22], and the solvent injection method [52, 53]. Also, different strategies like ultrasonication [22, 50] and membrane extrusion have been utilized to reduce the particle size of TPGS-liposomes for cancer delivery applications [54, 55].

## TPGS Liposomes for Anti-cancer Drug Delivery

Liposomes have been used to encapsulate several valuable chemotherapeutic drugs, including cisplatin, doxorubicin, paclitaxel, and methotrexate, to reduce their toxic side effects while maintaining or even improving efficacy [56]. Liposomal formulations can improve *in vivo* toxicity and efficacy profiles through modifying pharmacokinetics and biodistribution [57]. For example, encapsulation of doxorubicin into conventional or PEGylated liposomes led to better cardiac safety and less myelosuppression, alopecia, nausea and vomiting than conventional solution formulations [58]. Liposomes may also enhance the uptake of some encapsulated therapeutics into target cells, such as tumour cells, due to the similar membrane structure of liposomes and cells, leading to uptake into cells by endocytosis [42].

Encapsulation within liposomes has also been proposed to enhance chemotherapeutic delivery to tumours through the enhanced permeability and retention (EPR) effect. Still controversial, the EPR effect proposes that nano-carriers, including liposomes, experience enhanced permeation into and retention within tumours compared to healthy tissue. This is because the tumour is proposed to have malformed and ‘leaky’ blood vessels that are more permeable to nano-carriers than blood vessels in healthy tissues [59]. Further, removal of nano-carriers from the tumour may be slowed by impaired lymphatic function resulting from compression of the lymphatics in the rapidly growing tumour. However, studies have reported results both for and against the existence of the EPR effect, which is why it remains a controversial topic in drug delivery [60].

In the cancer setting, TPGS liposomes have thus been used to extend pharmacokinetics, improve uptake into target cells whilst reducing exposure to off-target tissues and, as a result, increase drug safety and efficacy. These beneficial effects result from the combination of using a liposome and surface modification with TPGS. TPGS modification on liposomes can, for example, prolong circulation half-life by avoiding opsonisation and uptake by phagocytic cells. Importantly, TPGS is also a potent P-gp inhibitor that can effectively reduce drug efflux from tumour cells. Several mechanisms have been proposed for this P-gp inhibition. First, binding of TPGS to the non-transport active site of P-gp may lead to a conformational change and disruption of the transport function of P-gp [61, 62]. Second, other studies suggested that TPGS can bind to the ATP active site of the P-gp nucleotide-binding domain. This blocks the binding of the ATPase, limits ATP hydrolysis, and subsequently disrupts the P-gp energy supply, reducing substrate efflux [63, 64]. Third, TPGS may inhibit P-gp by affecting mitochondrial function [65–67]. This phenomenon has been reported for TPGS-based nanocarriers. For example, Wang *et al.* reported that TPGS could affect mitochondrial function by disrupting the microenvironment of P-gp by damaging the mitochondrial double membrane structure and decreasing ATPase production [68]. Also, another study showed that TPGS could induce mitochondrial apoptosis, which helps inhibit P-gp in tumor cells [69]. Another report suggested that the MDR reversing effect of TPGS is mainly associated with inhibition of mitochondrial respiratory complex II, which reduces the supply of ATP to transporters [70–72].

TPGS on the outer surface of liposomes might also act as a ligand that directly interacts with membrane receptors, resulting in receptor-mediated endocytosis of the liposomes and enhanced uptake of chemotherapeutics into target cells [73, 74]. TPGS, therefore, has multiple potential effects that can benefit anti-cancer drug delivery. Below is a summary of specific studies demonstrating the successful application of TPGS-modified liposomes to treat different types of cancer.

## Brain Cancer

Brain tumours typically have a poor prognosis with an average life expectancy of approximately one year [75, 76]. Among brain cancer types, glioblastoma multiforme is a common tumour [77], with a mean survival rate of ~ 3.3% at three years post-diagnosis [77, 78]. Brain cancers have traditionally been treated with a surgical procedure followed by systemic chemotherapy, but these are limited due to the effects on the brain and severe toxicity. Nanocarrier-based strategies to enhance anti-cancer drug delivery into the tumor site within the brain without destroying any healthy tissues or organs are thus being widely studied [79].

The major challenge to chemotherapeutic delivery to the brain is the presence of the blood–brain barrier (BBB). The BBB is a protective barrier between the blood and brain tissue, preventing molecules from entering the central nervous system [80]. The BBB is formed through microvascular endothelial cells wrapped by tight junctions, adherens junctions, microvessels, pericytes, and astrocytes and interconnected by microglia and neurons [81, 82]. The BBB is an obstacle to transporting many therapeutic molecules into and out of the brain [83]. More than 90% of small molecules and approximately 100% of large therapeutics are prohibited from crossing the BBB [80]. Additionally, those few therapeutic agents capable of permeating the BBB may be actively transported back into the blood vasculature via efflux transporters, such as P-gp [84]. Furthermore, metabolic degradation in the brain may reduce the accumulation of drugs in the brain [85].

As a drug carrier, liposomes are considered an excellent candidate for crossing the BBB due to their structural resemblance with the lipid bilayer of the endothelial cell membrane [86]. The outer surface of liposomes can also be modified to extend their circulation time in the blood, thus enhancing the time available to penetrate the brain [87]. Previously, many researchers have described the potential for TPGS-modified nano-carriers to cross the BBB, such as micelles, polymeric NPs, solid-lipid NPs and liposomes [88–91].

The surface modification of liposomes with TPGS has also shown significant potential to boost BBB uptake via inhibition of P-gp [92]. For example, Vijayakumar et al*.* developed a resveratrol-loaded TPGS-coated liposomal drug delivery system for brain targeting. The anti-cancer potential of resveratrol solution, uncoated liposomes, and TPGS-decorated liposomes was investigated in C6 glioma cancer cell lines. The study demonstrated an excellent cellular uptake of TPGS-modified liposomes into C6 glioma cancer cell lines and higher AUC and plasma half-life of TPGS- resveratrol liposomes compared to resveratrol in uncoated liposomes or free solution in rats after intravenous injection [93].

**Fig. 4:**
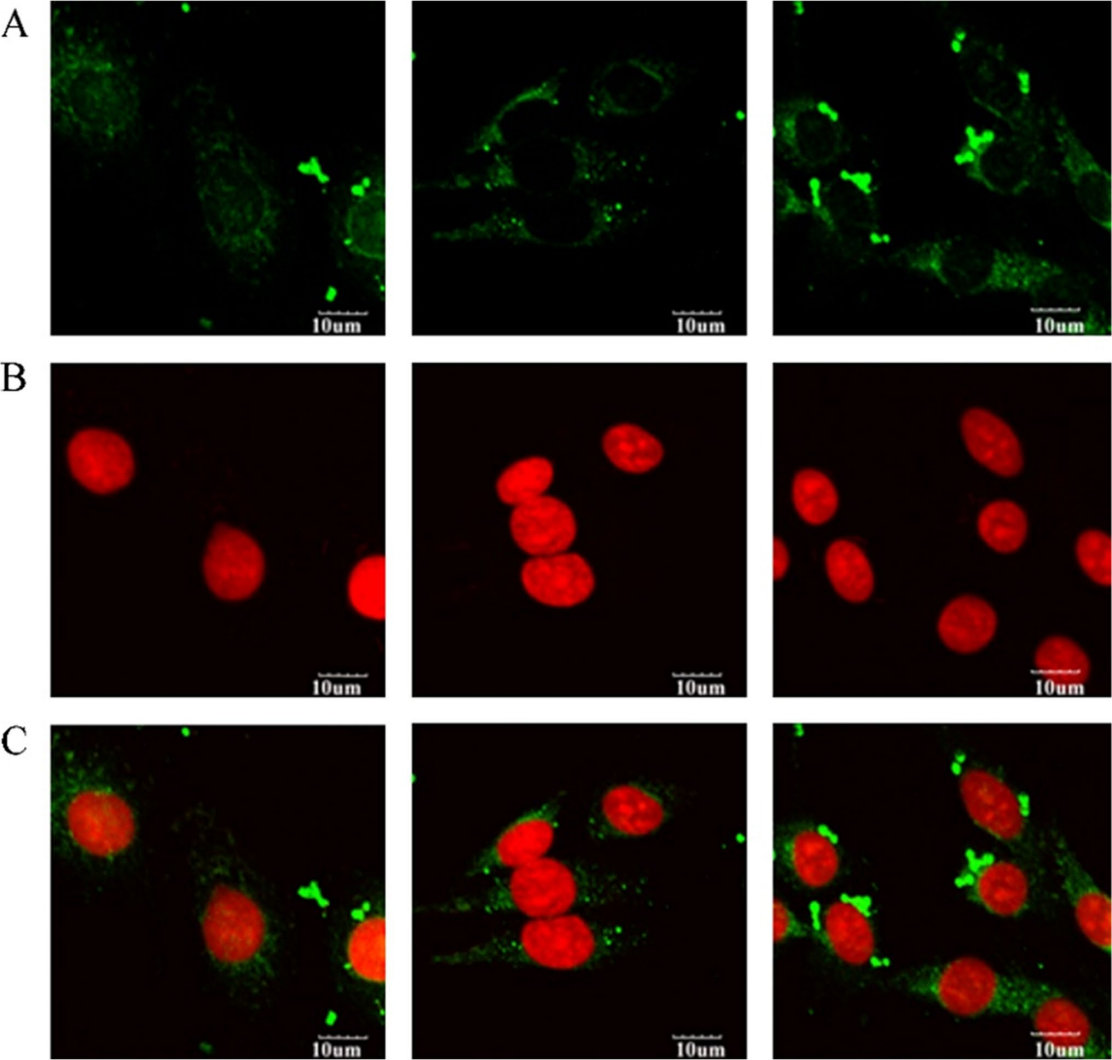
Confocal laser scanning microscopy (CLSM) of C6 glioma cells after 2 h incubation with fluorescent coumarin-6 loaded conventional liposomes (left column), PEG coated liposomes (middle column) and TPGS coated liposomes (right column). Row (**A**): fluorescein isothiocyanate (FITC) channel showing the green fluorescence from the liposomes distributed in the cell, (**B**): PI channels showing the red fluorescence from propidium iodide stained nuclei, and (**C**): merged channels of FITC and PI [53]. Reprinted and adopted with permission from Elsevier Ltd. through Copyright Clearance Centre.

Another study used TPGS-decorated liposomes to enhance docetaxel delivery to brain cancer cells. An *in vitro* study indicated that TPGS coating on liposomes might be a promising approach to enhance docetaxel delivery into C6 glioma cells [53]. The enhanced cellular uptake of TPGS liposomes (Fig. 4) as compared with conventional and PEG liposomes might be due to the TPGS coating on the liposome inhibiting P-gp expression and efflux [94].

Lin Lia *et al*. prepared docetaxel-loaded glutamate and TPGS functionalized liposomes to target the large amino acid transporter 1 (LAT1) to enhance BBB penetration and glioma therapy. LAT1 is a receptor abundantly expressed on the BBB and glioma cells. An *in vivo* investigation in mice showed that the modified liposomes significantly improved the BBB penetration compared to unmodified liposomal formulations [95]. The enhanced brain penetration may be partly due to the TPGS coating inhibiting P-gp-based drug efflux at the BBB, thus enhancing DTX concentration in the brain [96, 97].

In another approach, TPGS-liposomes were used for dual delivery of paclitaxel and artemether to treat invasive brain glioma. In this study, liposomes were prepared using two different functional materials mannose-vitamin E derivative conjugate and a dequalinium-lipid derivative conjugate. The liposomes successfully delivered both drugs across the BBB, destroyed vasculogenic mimicry channels in the brain tumour-bearing rats and enhanced *in vitro* apoptosis in glioma C6 cells [98].

Sonali *et al.* entrapped docetaxel and quantum dots (QDs) in the arginine-glycine-aspartic acid peptide (RGD) conjugated TPGS liposomes. An *in vitro* drug release study of the developed liposomes revealed that 80% of the drug was released from liposomes at pH 7.4 within 72 h. Next, uptake into the brain and the theranostic effect of liposomes on brain cancer were studied in rats. Results indicated that the targeted liposomes crossed the BBB and simultaneously delivered both the drug and QD imaging agent to brain tissue, leading to an enhanced theranostic effect in brain tumour [99]. The enhanced theranostic effect was possibly due to the internalization of RGD-decorated TPGS liposomes into the brain via integrin receptor-mediated endocytosis at the BBB [100].

## Breast Cancer

Breast cancer is the most commonly diagnosed cancer and is the leading cause of cancer-related death in women worldwide [101]. Breast cancer incidence and mortality rates are steadily increasing [102]. The milk ducts and inner linings of breast lobules are the primary sites for breast cancer initiation [103]. Currently, the treatment options for breast cancer therapy include surgery, radiation therapy and chemotherapy [104]. Numerous drawbacks are associated with these therapies, such as an inability to selectively target and kill tumour cells [105]. This has led to the widespread study of nanocarrier-based delivery approaches to improve breast cancer treatment [106, 107]. These nanocarrier-mediated approaches aim to deliver chemotherapeutic agents more directly to tumours leading to enhanced efficacy and reduced toxicity [108].

TPGS-modified liposomes have been used in the context of breast cancer to extend the blood circulation time of valuable chemotherapeutics and enhance drug targeting and accumulation in tumours [109]. Jin *et al.* designed a TPGS-modified liposome for co-delivery of paclitaxel with a newly approved tripeptide tyroservatide, a non-cytotoxic anticancer drug, to enhance treatment efficacy in breast cancer. The results of *in vitro* cellular uptake studies and *in vivo* animal experiments demonstrated that the surface-modified liposomes enhanced drug uptake into MB-231 breast cancer cells and increased delivery to tumours in MB-231 tumor-bearing nude mice following intravenous administration compared with free drug solution [110]. Another approach developed by Muthu *et al.* was a multifunctional TPGS liposomal drug delivery system containing docetaxel and QDs for cancer imaging and therapy. The TPGS-modified liposomes showed an excellent *in vitro* cytotoxic effect and enhanced cellular internalization in MCF-7 cells compared to non-modified liposomes [111].

Han and co-workers designed paclitaxel-loaded liposomes and TPGS-coated paclitaxel liposomes. Both liposomal formulations were spherical and exhibited sustained release of paclitaxel *in vitro* over 72 h at pH 7.4. Compared with non-coated liposomes, the TPGS-coated liposomes revealed higher *in vitro* cytotoxicity and cellular uptake in multidrug-resistant MCF-7/ADR cells [112]. The enhanced accumulation and cytotoxicity of TPGS-coated liposomes in MCF-7/ADR cells might be due to the inhibition of P-gp by TPGS [64]. Assanhou *et al.* further designed TPGS and hyaluronic acid coated dual-functional cationic liposomes to co-deliver paclitaxel and lonidamine and reverse MDR in cancer treatment. The TPGS coating and functionalization with hyaluronic acid enhanced the accumulation of paclitaxel and lonidamine at the tumour site, leading to higher anti-tumor efficacy in mice with a xenograft MCF-7/MDR tumour compared to non-coated liposomes [113].

Docetaxel-loaded TPGS liposomes have also been conjugated with the anti-human epidermal growth factor receptor 2 (HER2) monoclonal antibody, trastuzumab, which specifically binds to HER2-positive cancers such as breast cancers. In *in vivo* pharmacokinetic experiments, the TPGS liposomes exhibited a 1.9 and tenfold higher half-life than non-coated liposomes and a marketed docetaxel solution following intravenous administration in rats [52].

To enhance the *in vitro* cytotoxicity and cellular uptake of the histone deacetylase inhibitor, vorinostat, in breast cancer cells, the Wang group designed TPGS-coated vorinostat-loaded liposomes using thin film hydration with the probe sonication method [22]. An electron microscopy study confirmed the TPGS coating on the surface of liposomes. The TPGS-modified liposomes improved the solubility of vorinostat and sustained *in vitro* release of vorinostat over 48 h at pH 7.4 compared to non-modified vorinostat liposomes. Fluorescence microscopy and flow cytometry investigations showed higher cellular uptake of TPGS-coated liposomes into MCF-7 breast cancer cells than non-modified vorinostat liposomes suggesting their potential to provide better treatment of breast cancer [22].

## Lung Cancer

Lung cancer is one of the leading causes of death, with over 1.7 million deaths worldwide each year [114]. The inadequate efficacy and side effects of available traditional lung cancer chemotherapies are major hurdles for lung cancer patients. Nanocarrier-based approaches are ideal for lung cancer therapy [115–117], especially surface-modified liposomes, which enhance the solubility, and stability and improve the delivery of therapeutics to target organs and tissues for cancer therapy [118, 119].

Luteolin is a natural product widely used in various Chinese herbal medicines. In a study by Li *et al.,* TPGS-liposomes were fabricated to enhance the accumulation of luteolin in the lungs. The TPGS-coated drug-loaded liposomes improved apoptosis and enhanced cytotoxicity in A549 lung cancer cells compared to free luteolin. Also, the modified liposomes supported increased drug accumulation in the tumour tissues and improved tumour growth inhibition in A549 tumor-bearing nude mice compared to free drug solution [120]. Similarly, in another study, ginsenoside compound K was encapsulated in TPGS-decorated liposomes to enhance ginsenoside solubility and *in vivo* targeting. The TPGS-liposomes displayed slower *in vitro* drug release than free ginsenoside and excellent drug loading. Compared with the free drug solution, the TPGS liposomal formulation exhibited higher uptake into A549 lung cancer cells and enhanced *in vivo* antitumor efficacy in A549 lung cancer tumor-bearing athymic nude mice [121].

Li and his group also designed TPGS liposomes loaded with docetaxel to reverse MDR and boost lung cancer treatment. The TPGS-modified liposomes displayed dose and time-dependent cancer cell killing and improved intracellular drug accumulation in A549 lung cancer cells compared to the free drug solution. *In vivo* anti-tumor studies revealed that the TPGS-coated docetaxel-loaded liposomes had excellent anti-tumor efficacy in an A549/DDP xenograft mouse model compared to free drug [122].

## Liver Cancer

Liver cancer is the fifth most diagnosed cancer and the third leading cause of cancer deaths worldwide [123]. Hepatocellular carcinoma is a frequently occurring type of liver cancer and is hard to detect before the tumours are quite progressed, leading to increased treatment difficulty [124]. Nanocarrier-based approaches have allowed researchers to improve the efficacy of drugs against liver cancer. This is achieved through enhanced delivery to the tumour leading to a reduction in the drug dose required to produce a therapeutic effect and reduced systemic toxicity. Further, nanocarrier approaches can extend drug release to days following a single dose and boost the delivery of cancer therapeutics to liver cancer cells [125].

Dual delivery of anti-cancer drugs and small interfering RNA (siRNA) has been described as a potential novel strategy for enhancing the efficacy of anti-cancer drugs and overcoming MDR [126]. As an example, Xi Tan and his team designed a TPGS liposomal co-delivery system containing doxorubicin and Bcl-2 siRNA to achieve a synergistic effect and enhance the anti-cancer efficacy of doxorubicin in H22 tumor-bearing mice [127]. Dual Bcl-2 siRNA and doxorubicin-loaded TPGS-coated liposomes significantly enhanced the cellular uptake of doxorubicin into H22 cells compared to doxorubicin in unmodified liposomes. There was also prolonged blood circulation and higher doxorubicin accumulation at tumour sites in H22 tumor-bearing mice after administration of the TPGS coated compared to uncoated liposomes [127]. The higher accumulation of TPGS-coated liposomes at tumour sites than non-coated liposomes was possibly due to the PEG layer on the TPGS-coated liposomes leading to modified clearance and tissue disposition [128].

Yinghuan Li *et al.* designed a co-delivery strategy to reverse MDR and treat hepatocellular carcinoma using Bcl-2 siRNA and doxorubicin-loaded TPGS-coated cationic liposomes. This study demonstrated that the TPGS-liposomes encapsulating doxorubicin and siRNA extended the intracellular doxorubicin retention time in Bel7402 or Bel7402/5-FU cells and the siRNA containing liposomes enhanced the internalization of doxorubicin into Bel7402 and Bel7402/5- FU MDR cells as compared with standard doxorubicin liposomes [129]. However, siRNA-loaded liposomes also improve doxorubicin uptake from TPGS-liposomes, which may be due to the size-dependent uptake of siRNA-loaded cationic liposomes [130, 131]. Cheng Hu and his team also developed artesunate-TPGS liposomes to enhance drug stability and liver targeting to treat liver cancer. An *in vivo* pharmacokinetic investigation showed that TPGS-coated liposomes increased the plasma drug concentrations and prolonged the circulation half-life of artesunate compared to a free solution of artesunate injected through an intravenous route in rats [132].

## Blood Cancer

Leukaemias are hematologic malignancies that frequently occur in young adults and mainly affect the bone marrow, lymphatic system, and immune cells [133]. In 2020, approximately 0.437 million new cases and 0.309 million cancer deaths were reported from leukemia globally [134]. The current treatment options for leukemia are usually chemotherapy, radiation therapy and bone marrow transplantation [135]. The current therapies are associated with off-target side effects, so nanomedicines may provide an approach to enhance the efficacy and toxicity profiles of various anticancer drugs against leukemia [136].

Previously methoxy polyethylene glycol 2000-derivate distearoyl phosphatidylethanolamine (mPEG2000-DSPE) has been used for the surface modification of liposomes to enhance the stability and extend their circulation time in blood. mPEG2000-DSPE has PEG with a molecular weight of 2000 Da, which may reduce interactions of the liposomal formulation with target leukaemia cells and may be associated with side effects such as skin toxicity or immunogenicity from its prolonged circulation time in the blood [137]. TPGS,, which typically has a shorter chain of PEG with 1000 Da compared to mPEG2000-DSPE, may be used as an alternative PEGylating agent to enhance delivery to leukaemia cells [138, 139].). In the literature, replacement of PEG by TPGS for surface modification of liposomes in one case resulted in a longer circulation time [139].

As an example, Wang *et al.* encapsulated emodin, a multifunctional traditional Chinese drug with low water solubility, into TPGS liposomes. The resulting liposomes showed a high drug encapsulation efficiency of 95.2 ± 3.0%, mean particle size 121.1 ± 44.9 nm, spherical shape, and sustained release profile. The TPGS-coated liposomes improved the *in vitro* cytotoxicity of emodin against L1210 and K562 leukemia cell lines. *In vivo* results indicated increased plasma half-life of emodin following intravenous administration in TPGS liposomes compared with free drug solution in rats [55].

## TPGS Liposomes for Other Applications

### Oral Bioavailability Enhancement

Many drug delivery systems have been designed to boost the oral delivery of poorly water-soluble drug molecules, including polymeric micelles, nanosuspension, solid dispersions, lipid-based formulations and liposomes [140]. Among these nanocarriers, liposomes are ideal for enhancing the solubility of drugs in an aqueous environment and thus boosting drug absorption capacity following oral delivery [141]. Poorly water-soluble lipophilic drug molecules can be solubilised within the phospholipid bilayer of liposomes [142, 143]. A major challenge, however, to the oral delivery of liposomes is inadequate stability during passage through the gastrointestinal tract. For example, pH changes from the stomach to the intestine coupled with the presence of lipid digestive enzymes can lead to a breakdown in the liposome structure and premature drug release [144]. Nonetheless, several studies have reported that TPGS liposomes can boost the absorption of drugs following oral delivery.

Liu *et al.* designed TPGS-decorated liposomes loaded with isoliquiritin to enhance isoliquiritin oral bioavailability and liver targeting, both major hurdles to its therapeutic application. Isoliquiritigenin is a flavonoid compound mainly obtained from the root of *licorice* [145]. Past studies have demonstrated the anti-cancer activity of isoliquiritigenin *in vitro* against various liver cancer cell lines, including HepG2 and Bel7402 cells, and *in vivo* in liver cancer models [146, 147]. However, the clinical application of isoliquiritigenin is limited by low aqueous solubility, leading to poor absorption and oral bioavailability and poor targeting of the liver [148]. In the study by Liu *et al.,* TPGS-decorated isoliquiritin liposomes were fabricated using the thin-film dispersion technique. Following oral delivery, the TPGS-decorated isoliquiritin liposomes enhanced the area under the plasma concentration–time curve (AUC_0_-24 h), and thus oral bioavailability of isoliquiritin was 1.53-fold compared to a drug suspension. A tissue distribution study also revealed a higher accumulation of isoliquiritin in the liver following oral administration in TPGS liposomes compared to the free isoliquiritin solution in ICR (institute of cancer research) mice [24]. The enhanced oral bioavailability and liver targeting of isoliquiritin-TPGS liposomes may be due to TPGS liposomes enhancing drug solubilisation and thus absorption. TPGS may also potentially inhibit P-gp mediated efflux, leading to enhanced intestinal absorption and accumulation in the liver [149]. Also, TPGS-coated liposomes may potentially improve drug stability *in vivo* via inhibition of CYP3A4 and CYP2C9 mediated metabolism [150].

An attempt was also made to improve the oral bioavailability of syringic acid through administration in TPGS-coated liposomes. Syringic acid is a natural phenolic acid present in plants and foods such as black olive, walnut, and cinnamon [151]. Previously syringic acid has been used as a broad-spectrum antibiotic, and recent studies have shown its anti-cancer effect against skin cancer [152]. But, syringic acid is subject to rapid renal excretion and low oral bioavailability due to poor water solubility, which reduces efficacy after oral dosing [153]. In a study by Li *et al.,* syringic acid-loaded TPGS liposomes were prepared with spherical morphology and encapsulation efficiency of 96.48 ± 0.76%. Pharmacokinetic studies showed that TPGS-coated liposomes delayed renal elimination of syringic acid and significantly increased liver concentrations of the drug in rats. The TPGS-liposomes also increased the oral bioavailability of syringic acid 2.8-fold compared with the administration of a free drug [154]. This was attributed to the addition of TPGS to the liposomes acting as a permeation enhancer leading to improved drug permeation across the intestinal wall [155].

6-shogaol is a component of ginger with multifaceted therapeutic potential, including anti-cancer and antioxidant activities. However, its lower solubility in water and, thus, low oral absorption have limited its clinical application [156]. So, Rui Bao *et al.* prepared 6-shogaol in TPGS-coated liposomes to enhance water solubility and oral bioavailability. The TPGS modified liposomes and conventional liposomes had a relative oral bioavailability of 580.04% and 281.55%, respectively, compared to free drug [157]. The TPGS-modified liposomes also enhance the brain accumulation of 6-shogaol 2.00 and 4.62-fold compared to conventional liposomes and free drug solution, respectively, at 1 h following administration [157]. The increased brain accumulation was proposed due to the coating of TPGS on the liposome, inhibiting p-gp mediated drug efflux across the BBB and improving drug accumulation in the brain [158, 159].

### Ocular Delivery

For ocular delivery, liposomes can enhance the solubilization of hydrophobic drugs [160] and increase corneal drug permeability by providing close contact with the cornea, conjunctiva, and prolonged corneal contact time [161]. Many PEG and TPGS-based nanocarriers have been designed for ocular delivery of therapeutic molecules, including NPs [162, 163], micelles [164] and solid lipid NPs [165]. The TPGS-modified nanocarriers may enhance the trans-corneal permeability of ocular drugs either passively or by reducing P-gp mediated efflux resulting in improved drug absorption [166]. TPGS-modified nanocarriers could also enhance drug stability, which prevents drug loss from formulations [167].

**Fig. 5:**
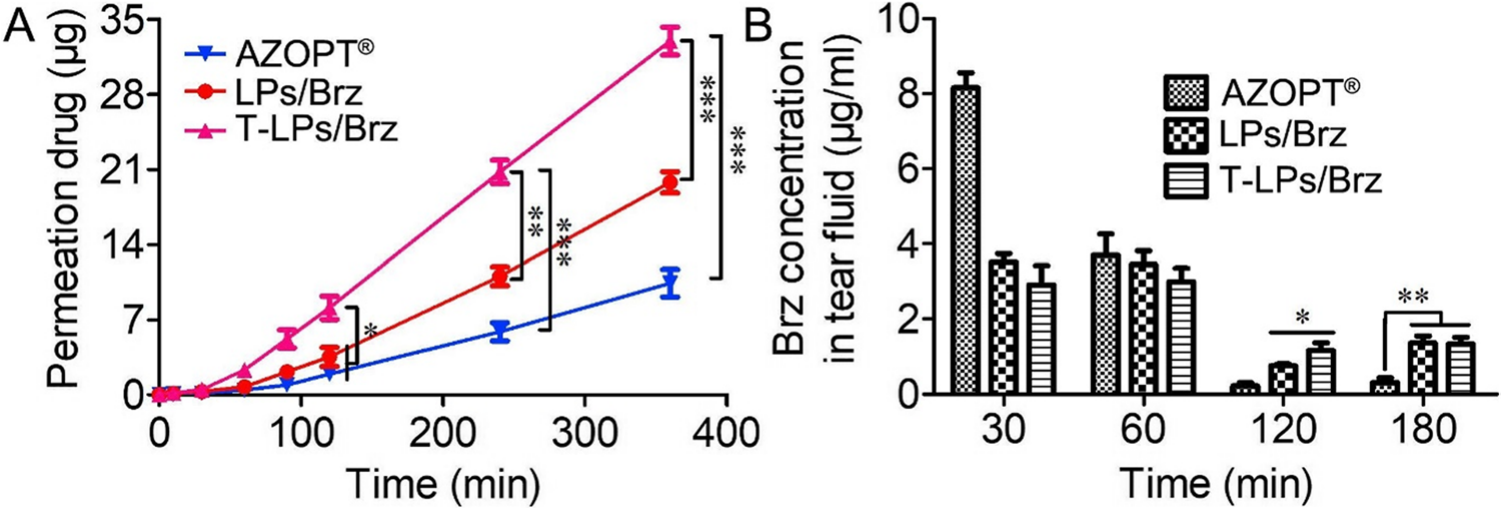
**A** Corneal permeation of brinzolamide after *in vitro* application in TPGS liposomes (T-LPs/Brz), conventional liposomes (LPs/Brz) or the marketed Azopt® aqueous suspension formulation**. B** Brinzolamide (Brz) concentration in tear fluid at different times following application in TPGS liposomes (T-LPs/Brz), conventional liposomes (LPs/Brz) or the marketed Azopt® aqueous suspension formulation. All data is expressed as mean ± SD for n = 3. *P < 0.05, **P < 0.01, ***P < 0.001. Reprinted with permission from Elsevier Ltd. through Copyright Clearance Centre.

As an example, Quansheng Jina and co-workers designed TPGS-modified nano-liposomes to deliver brinzolamide to the eye for glaucoma therapy. The TPGS-modified liposomes provided higher encapsulation efficiency (over 90%) and more sustained drug release compared to conventional liposomes. Additionally, brinzolamide-TPGS liposomes enhanced *in vitro* penetration of brinzolamide across rabbit cornea 2 and 5 times higher than conventional liposomes and the marketed Azopt® suspension formulation, respectively (Fig. 5A). The concentration of brinzolamide in the tear fluid was also studied for the three formulations, with no difference seen at 60 min but at 120 min the TPGS-liposomes showed the highest concentration. TPGS-liposomes also showed low toxicity with around 100% cell viability of L929 cells and no significant side effects in an animal model after treatment with the coated liposomes**.** TPGS-liposomes might therefore be a promising carrier for efficient delivery of therapeutics for glaucoma therapy [168].

### Vaccine Delivery

For vaccine therapy, the delivery of antigen to the correct target cells in the immune system is important to maximise response. Typically the vaccine antigen needs to be delivered to antigen-presenting cells (APCs), such as dendritic cells or macrophages, which present the antigen to B and T cells leading to the generation of immunity toward the specific antigen [169]. The B and T cells are concentrated in lymph nodes throughout the body, and typically antigen is picked up by APCs either at the injection site or in the lymph node draining the administration site and from here, the APCs make their way to the B and/or T cell zone of lymph nodes to present the antigen. Further, numerous studies have reported that prolonged antigen persistence may enhance immune responses [170]. Nanomedicine scientists have thus developed nanocarrier delivery systems to enhance and control the delivery of antigens and adjuvants to lymph nodes and target immune cells [170].

Several nano-sized drug delivery systems have been applied for vaccine delivery, including microemulsions, nanoparticles and liposomes [171, 172]. These vaccines have been administrated through various routes, including oral, intranasal, intramuscular, intradermal and subcutaneous injections. The properties of nanocarriers, such as size, shape, surface charge, surface modification etc., can all impact the rate of absorption from administration sites, uptake into lymphatics, and delivery to immune cells. Recent reviews have highlighted these factors [173–175]. Of note, a diameter of 10–150 nm may optimise delivery to the draining lymph node as smaller particles are typically absorbed directly into draining blood vessels (which have > 100-fold higher flow of blood compared to lymph flow), whereas larger particles may become entrapped at injection sites. A size of 10–150 nm can lead to specific transport via the lymphatic vessels to lymph nodes as the initial lymphatics have open button-like junctions that enable the entry of particles, whereas the closed tight junctions present in blood capillaries typically hinder the entry of particles in this size range. Nanomedicine-based vaccines may utilise these principles to enhance delivery to lymph nodes and immune cells [173].

The properties of liposomes such as size, surface charge and PEGylation are also important in controlling local tissue distribution, retention, trafficking, uptake and processing by APCs in the lymph nodes [176]. In the vaccine delivery system, the surface of liposomes can be modified to improve the immune response to weakly immunogenic protein antigens or synthetic peptides [177]. For example, PEG coatings may enhance the immune response when conjugated with other entities such as proteins and peptides [178]. In this way, modified liposomes can elicit a more robust immune response than conventional liposomes [179].

**Fig. 6:**
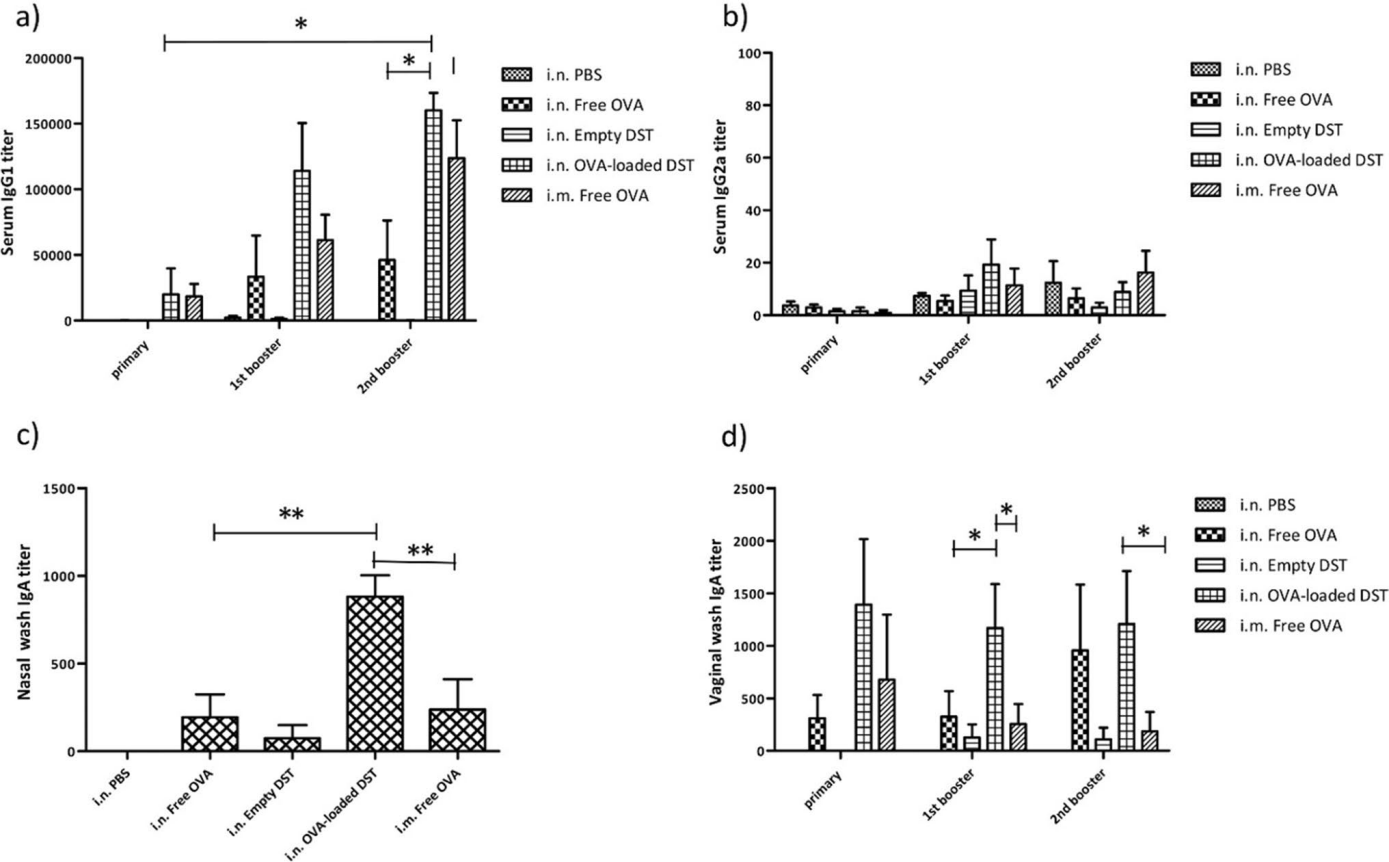
**a** OVA-specific serum IgG1 titers after vaccinations. Data are presented as mean ± sem of n = 4 to 5. *p < 0.05; **b** OVA-specific serum IgG2a titers after vaccinations. Data are presented as mean ± sem of n = 4 to 5; **c** OVA-specific IgA titers from vaginal wash after vaccinations. Data are presented as mean ± sem of n = 4 to 5. *p < 0.05; **d** OVA-specific. IgA titers from nasal wash at day 42. Data are presented as mean ± sem of n = 4 to 5. **p < 0.01 [181]. i.n. = intranasal, i.m. = intramuscular, DST = TPGS coated liposomes. Reprinted and adopted with permission from Elsevier Ltd. through Copyright Clearance Centre.

TPGS is an alternative approach to coat the surface of liposomes with PEG to enhance vaccine efficacy, although few studies have evaluated this to date [180]. As an example, Yusuf et al*.* designed a needle-free mucosally delivered vaccine using TPGS-coated ovalbumin-loaded freeze-dried liposomes. TPGS-liposomes were in the nano-size range and stable in terms of particle size. In an *in vivo* study, the TPGS-liposome-loaded ovalbumin provided a strong immune response in mice following intranasal administration compared to a free ovalbumin solution (Fig. 6). [181].

## Conclusion and Future Perspectives

Liposomes are multipurpose nanocarriers used for a range of applications in drug delivery. TPGS is a biocompatible material that is readily available on a commercial scale. TPGS has been widely used in drug delivery due to desirable properties such as nontoxicity, biocompatibility, and biological safety in addition to enhancing emulsification, solubilisation, permeability etc. The surface of conventional liposomes can be modified via the addition of TPGS. This TPGS coating can prolong circulation half-life by facilitating avoidance of opsonisation and phagocytosis by the reticulo-endothelial system. Further, TPGS coating may enhance the cellular uptake of liposomes both by increasing incorporation into the lipid membranes and through inhibition of efflux by transporters such as P-gp. These desirable properties of TPGS-coated liposomes have led to their application to enhance the treatment of various cancers, including breast, liver and brain cancer. For cancer therapy, TPGS-decorated liposomes have improved the efficacy and toxicity profile of poorly soluble anticancer drugs through changes to the pharmacokinetics, biodistribution and enhanced uptake into target cells. Animal studies have indicated no side effects associated with TPGS-based liposomes compared to conventional liposomes. In other applications, TPGS-based liposomes have been shown to benefit oral, ocular and vaccine delivery through improving drug stability, solubility, delivery, pharmacokinetics and the vaccination effect.

In past studies, most TPGS-coated liposomes were loaded with a single therapeutic agent, and only a few studies have described loading multiple drugs and/or imaging agents for cancer treatment, diagnosis and targeted delivery. We recommend that TPGS-liposomes are also an excellent carrier for encapsulating multiple drugs and imaging agents for targeted drug therapy and imaging. In the future, TPGS-modified liposomes may thus be used for theranostic purposes by encapsulating a combination of therapeutic and diagnostic agents. TPGS-coated liposomes offer many potential benefits for the delivery of different therapeutic molecules and may therefore be explored for application in other disease states in future, such as inflammatory and infectious diseases.

Although TPGS liposomes show remarkable potential for the treatment of various cancers and other diseases, many challenges remain to be solved. The underlying mechanisms by which liposomes enhance uptake into and treatment of cancers are not completely clear and should be investigated for a more comprehensive application of TPGS-liposomes to treat tumours and metastases. TPGS-liposomes can alter the pharmacokinetics and biodistribution of encapsulated molecules, but further studies could usefully determine the impact of the concentration and arrangement of the TPGS coating on the liposome on pharmacokinetics in more detail. The attachment of targeting ligands, antibodies, and aptamers to the surface of TPGS-liposomes to achieve targeted delivery is also an area ripe for further exploration, particularly to enhance the efficacy and toxicity of anti-cancer therapies. Most importantly, there is a need for a systematic evaluation of the impact of different size, surface charge and routes of administration on the biodistribution and efficacy of TPGS-liposomes, particularly for vaccine delivery, where delivery to immune cells in the lymph node is important. Since TPGS liposomes can be used as adjuvants in vaccine development for cancer immunotherapy and infectious diseases, the effects of TPGS on the immune system should be investigated in more detail.

Additionally, the development of TPGS-modified liposomes is still at the laboratory scale. The development of these liposomes is relatively slow, hindering successful clinical translation of TPGS-based liposomes. Optimizing the process of TPGS-liposome manufacture for large-scale industrial production while minimizing costs and maintaining control of the physicochemical properties of the TPGS-liposomes will be imperative to clinical translation. Studies on the stability of TPGS-decorated liposomes during storage are also essential, in addition to investigating the biological fate of formulation components and their long-term effects on human patients.

Overall, TPGS-modified liposomes are a promising drug delivery system to enhance the pharmacokinetics and therapeutic effect of a range of different molecules. We hope to see the TPGS-liposome products used in the clinic in the near future.

## Abbreviations

(APCs): Antigen-presenting cells
(BBB): Blood–brain barrier
(CMC): Critical micelle concentration
(EPR): Enhanced permeability and retention
(FDA): Food and Drug Administration
(HER2): Anti-human epidermal growth factor receptor 2
(HLB): Hydrophilic-lipophilic balance
(LAT1): Large amino acid transporter 1
(MDR): Multidrug resistance
(NPs): Nanoparticles
(PEG): Polyethylene glycol
(P-gp): P-glycoprotein
(QDs): Quantum dots
(RGD): Arginine-glycine-aspartic acid peptide
(siRNA): Small interfering RNA
(TPGS): D-alpha-tocopheryl polyethylene glycol succinate
